# A potential role for insulin treatment during pregnancy in reducing postpartum psychological distress in maternal obesity: an administrative population health study

**DOI:** 10.1186/s12905-021-01261-0

**Published:** 2021-03-20

**Authors:** Jessica S. Jarmasz, Alexandrea Anderson, Margaret E. Bock, Yan Jin, Peter A. Cattini, Chelsea Ruth

**Affiliations:** 1 Department of Physiology and Pathophysiology, Rady Faculty of Health Sciences, Max Rady College of Medicine, University of Manitoba, 419-745 Bannatyne Avenue, Winnipeg, MB R3E 0J9 Canada; 2 Department of Community Health Sciences, Rady Faculty of Health Sciences, Max Rady College of Medicine, University of Manitoba, 408-727 McDermot Avenue, Winnipeg, MB R3E 2P5 Canada; 3 Department of Pediatrics and Child Health, Rady Faculty of Health Sciences, Max Rady College of Medicine, University of Manitoba, 408-727 McDermot Avenue, Winnipeg, MB R3E 2P5 Canada

**Keywords:** Maternal obesity, Postpartum psychological distress, Insulin, Gestational diabetes, Mood and anxiety disorder, Pregnancy, Human placental lactogen, Administrative data, Epidemiology

## Abstract

**Background:**

Studies have found an association between obesity and an increased risk for peripartum depression, which has also been linked to decreased placental lactogen levels. In addition, women with obesity treated for gestational diabetes with insulin were found to have increased levels of placental lactogen. Treatment options exist for perinatal and postpartum depression however they pose a risk to the developing offspring. Thus, prevention as well as markers for early identification of peripartum depression are needed. Therefore, our study objective is to identify the association between insulin treatment in pregnancy and the risk of postpartum psychological distress (abbreviated here as PPD) among cohorts of women with and without obesity.

**Methods:**

Administrative health data (2002/03–2018/19) were used to identify a cohort of women (age 15+ years) who gave birth (N = 250,746) and had no pre-existing mood/anxiety disorders or diabetes (N = 222,863 excluded). Women were then divided into two groups: lean (N = 17,975) and with obesity (N = 9908), which was identified by a recorded maternal weight of > 38 to < 65.6 kg and ≥ 85 to < 186 kg (respectively). The risk of PPD within one year after delivery with and without insulin treatment was assessed by Poisson regression analysis. Models were adjusted for maternal age group (at pregnancy start date) and area-level income (at delivery).

**Results:**

The unadjusted risk of PPD was higher in the obesity group (8.56%; 95% CI 8.00–9.15) than in the lean group (6.93%; 95% CI 6.56–7.33). When no insulin treatment was given during pregnancy, mothers with obesity had a significantly higher risk of PPD than the lean group (aRR 1.27; 95% CI 1.17–1.39; *p* < 0.0001). However, when women with obesity and insulin treatment were compared to the lean group with no insulin treatment, no significant difference in the risk of PPD was observed between the groups (aRR 1.30; 95% CI 0.83–2.02; *p* = 0.248).

**Conclusion:**

This is the first study to demonstrate a positive association between insulin treatment in pregnancy among women with obesity and reduced PPD rates, suggesting insulin as a possible preventative measure. However, the biological mechanism behind the observed positive effect of insulin on PPD rates remains to be investigated.

**Supplementary Information:**

The online version contains supplementary material available at 10.1186/s12905-021-01261-0.

## Introduction

In 2017, 25.0% of Canada’s female population aged 18–34 years were considered overweight and 19.3% were considered to have obesity [1]. Pregnant women with obesity are at greater risk for developing gestational *diabetes mellitus* (GDM) or gestational hypertension (with or without proteinuria) [2–4], putting the fetus at risk for premature birth, caesarian section delivery, macrosomia, birth defects, premature death, and stillbirth [3, 4]. A 2017 systematic review suggested that women with obesity are at increased risk for antenatal depression [5]. There is a strong association with pre-pregnancy obesity and screening positive for postpartum depression [6, 7]; and it is estimated that 10–13% of women will develop a depressive episode postpartum [8]. Peripartum depression, which refers to a major depressive episode occurring during pregnancy or within 4 weeks following delivery, is observed in 8–13% of mothers with negative effects on the mother during and after pregnancy and on the offspring [9]. Despite the seriousness of the condition, it is difficult to diagnose and treat depression both during and shortly after birth [10, 11]. Thus, it is necessary to identify markers and potential therapeutic targets for early diagnosis, treatment and prevention of postpartum depression.

Pregnancy is a dynamic condition involving systemic changes in the mother that are largely mediated by the placenta—an organ that produces and secretes hormones to support pregnancy and fetal growth [12, 13]. Several hormones are involved in the physiological and neurological changes that occur in the mother in response to pregnancy, some of which are steroids (e.g., estrogens, progesterone), neuropeptides (e.g., oxytocin) and lactogenic hormones (e.g., prolactin (PRL), placental lactogen) [13]. Placental lactogens (PLs; also known as chorionic somatomammotropin (CS)) hormone, bind the PRL receptor with high affinity in order to mediate their effects in the body and brain [14, 15]. PL also binds the growth hormone receptor, although with much lower affinity [15]. Metabolically, PLs support pregnancy by increasing insulin production through expansion of pancreatic β-cells. Together with human placental growth hormone, this helps offset the effects of insulin resistance that normally occurs in pregnancy [16], and facilitates maternal energy production by metabolizing fats instead of carbohydrates (i.e., glucose) which are alternatively being delivered to the growing fetus [15, 16]. In the case of obesity, there is a surplus of fats and glucose in the body which leads to increased insulin resistance. This in turn can result in fetal hyperglycemia *in utero* and macrosomia at birth [17]. Equally so, certain neurological changes in the mother must also occur in order to facilitate appropriate and proper care for her newborn [13]. In two recent studies, PL was linked to depression [18, 19]. In the first study [19], placental samples from women with clinically diagnosed prenatal depression as well as in those self-reporting significant symptoms of depression during pregnancy were found to have a significantly decreased level of PL ribonucleic acid (RNA) [19]. In the second study [18], maternal serum PL taken just before birth was negatively associated with depression and anxiety scores assessed 10 weeks after birth. Interestingly, this observation was only seen among mothers who gave birth to girls. The authors also found a statistically significant relationship between PL serum levels and maternal body mass index (BMI) [18].

Previously, our research group conducted a study using placental samples taken at the time of birth from three groups of women: with obesity (BMI of 40.1 ± 1.6), with obesity and GDM treated with insulin (BMI of 38.7 ± 1.7), and without obesity (“lean”; BMI of 22.5 ± 0.5) [20, 21] in order to measure the effect of maternal obesity on PL levels in both tissue and in maternal serum. We reported a significant decrease in both PL RNA levels in placenta and serum PL levels in pregnant women with obesity at 28 weeks gestation [21]. When including mothers with obesity who received insulin during pregnancy to treat GDM, insulin treatment increased placental PL RNA and protein levels, meeting and exceeding the levels detected in the otherwise healthy lean group of women [21]. In another independent study, levels of PL were measured at birth in placentas from three groups of women: GDM controlled by diet, GDM controlled by glyburide or insulin, and BMI-matched controls [22]. BMI values ranged from 26.3 to 32.7, classifying these women as overweight/class I obese. Among the GDM controlled by glyburide/insulin, there were significantly higher PL levels in comparison to GDM controlled by diet and BMI-matched controls [22]. In a recent comprehensive review, decreased PL levels have been suggested to be a marker for maternal obesity, while increased levels is associated with maternal diabetes [23]. In addition, several studies have also linked obesity in pregnancy and GDM to the development of peripartum depression, both antenatal and postpartum [24–27].

Taken together, we hypothesize that: 1) maternal obesity, estimated as women weighing ≥ 85 to < 186 kg (with obesity group) versus those without (> 38 to < 65.6 kg; lean group), is associated with an increased rate of postpartum psychological distress; and 2) insulin treatment given during the gestational period will result in a reduced rate of postpartum psychological distress (abbreviated here as PPD) in women with obesity. Therefore, our objectives are to first, identify the risk of PPD among women with and without obesity, and second, observe the effect of insulin treatment during pregnancy on the risk of PPD. This will be effectuated utilizing population health data housed at the Manitoba Centre for Health Policy.

## Methods

### Data sources

The Manitoba Population Research Data Repository housed at the Manitoba Centre for Health Policy (MCHP; University of Manitoba, Canada) contains de-identified personal information about Manitoba residents enrolled in the Provincial health plan which provides coverage of insured health services for virtually the entire population of Manitoba [28]. Each individual is assigned a unique, nine-digit Personal Health Identification Number (PHIN) and an encrypted version of this PHIN is attached to person-level records in the Repository data files. This permits linkage of an individual across several data sources and years while still maintaining complete confidentiality. The Repository itself contains data collected by a variety of local agencies in Manitoba which have been previously validated for population-based research [29]. Nine data sources housed in the Repository were accessed for this study: Manitoba Health Insurance Registry (maternal age and insurance coverage), Canada Census File (area-level income quintiles), Drug Program Information Network (DPIN; prescriptions filled), Hospital Abstracts (delivery and birth records, mood/anxiety disorders, diabetes, postpartum psychological distress), Medical Services (mood/anxiety disorders, diabetes, postpartum psychological distress), Diagnostic Services of Manitoba (DSM; diabetes), Manitoba Diabetes Education Resource for Children and Adolescents (DERCA; diabetes), and the Manitoba Maternal Serum Screening Program (MMSSP; maternal pregnancy weights).

### Cohort formation

**Fig. 1:**
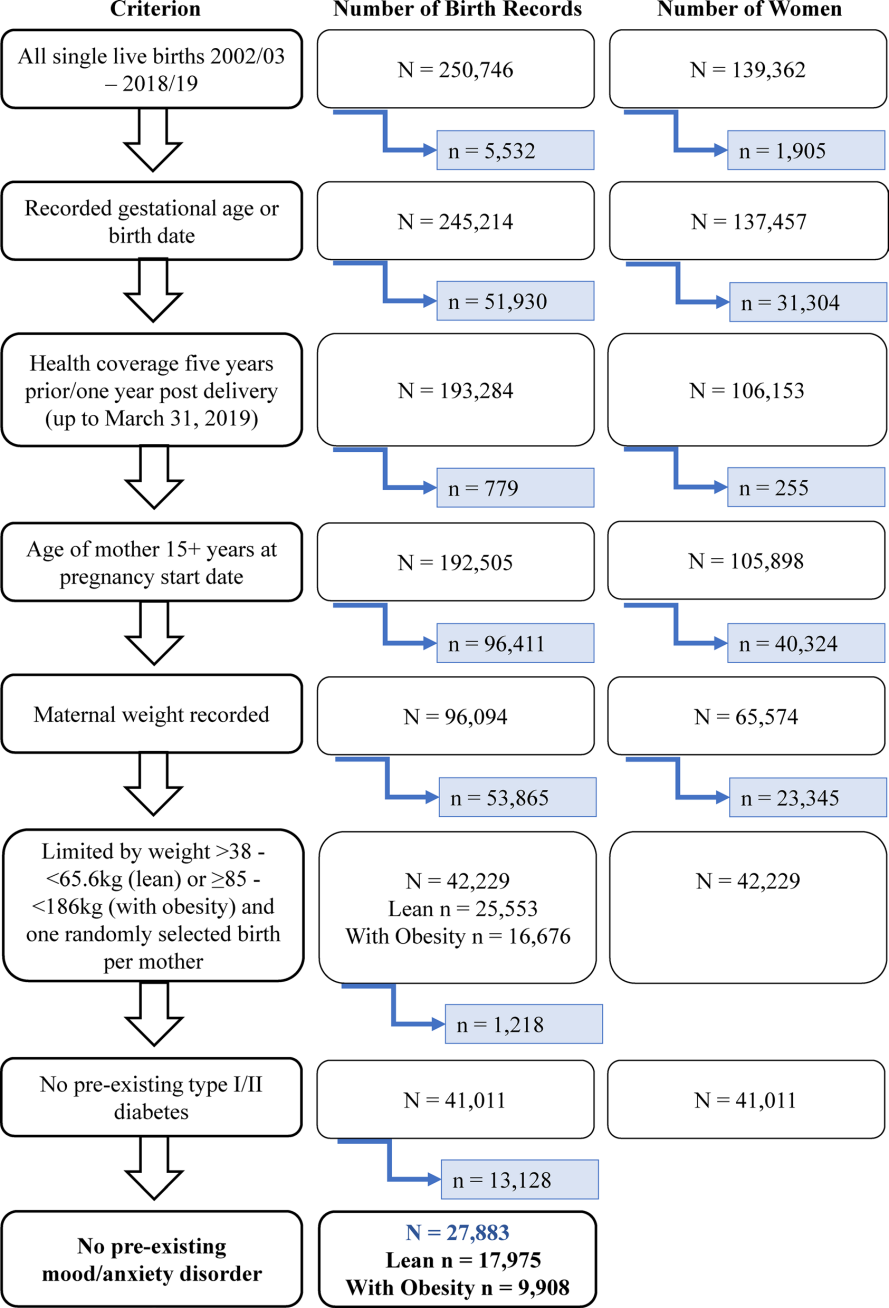
Flowchart showing the selection criteria, including the number (N) of birth records and women used to identify each of the two weight-based cohorts of women (lean and with obesity) to be assessed for risk of developing postpartum psychological distress with and without insulin treatment during gestation. Blue boxes represent the number of birth records and women excluded at every step of the cohort development

Hospital Abstracts were used to identify all records where a woman (N = 139,362) had an in-hospital single live birth between April 1, 2002 and March 31, 2019 (N = 250,746) (Fig. 1). The Hospital Newborn to Mother Link Registry (nblink) was then used to link the mother’s delivery record and the child’s birth record. This allows for pregnancy start date calculation and gestational period identification, based on the difference between the gestational age in weeks and the child’s birth date or, if missing, the mother’s hospital separation (discharge) date. Women with a missing pregnancy start date were excluded (n = 5532 excluded) (Fig. 1). In order to assure proper data capture, births were then limited to those where the mother had continuous health coverage under Manitoba Health from five years before their pregnancy start date up to one year following their delivery date (up to March 31, 2019; n = 51,930 excluded) (Fig. 1). Births were also limited to those where the mother’s age was 15 years or greater at the pregnancy start date (n = 779 excluded) (Fig. 1); an age of 15 years or greater was determined in order to apply a previously validated mood/anxiety disorder definition (see *Primary outcome* below). This gave a total of N = 192,505 births to N = 105,898 mothers (Fig. 1).

Manitoba Maternal Serum Screening Program Data were utilized to determine the mother’s weight during the gestational period where a first trimester and/or a second trimester weight is recorded. First trimester weight was selected however if missing, the second trimester weight was used. Weight ranges to classify women with and without obesity were chosen initially by using average female heights and weights reported by the Centers for Disease Control and Prevention (CDC) [30], and choosing weights that would reflect traditional BMI values used in studying weight classes in the “average population”. In 2013–2014, American women aged 20 years or greater weighed on average 169.8 lb (77.0 kg) and were 1.616 m tall (63.6 inches; 5 feet 3 inches), which translates into a BMI of 29.5 kg/m^2^ (overweight). Based on that value, weight cut-offs were determined based on a height of 64 inches (± 4 inches). For a BMI of 22 (lean/no obesity), the estimated weight range was 51.0–65.6 kg. For a BMI of 35 (with obesity; class II), the estimated weight range was 81.2–104.3 kg (see Additional file 1 for details). Therefore, in an effort to appropriately capture all women and avoid misclassification, we chose a recorded maternal weight range of > 38 to 65.6 kg to represent women in the lean group (without obesity), and a weight range of ≥ 85.0 to 186.0 kg for the obesity group (with obesity). These weight cut-offs were initially validated with pre-pregnancy height, weight and BMI information taken from a cohort of women (n = 62) classified as lean (n = 23) and obese (n = 39) in two previous studies of ours that gave us a capture rate of 90% for lean and 100% for obese groups (respectively; see Additional file 1 for details) [20, 21]. These weight cut-offs were then validated against a large 2017 publicly available dataset from the United States of female weights which also contained height, thus permitting BMI’s to be calculated [31]. Use of the same weight ranges in this dataset resulted in 9% of women misclassified as having obesity (who were not), and 0% of women misclassified as lean (who were not) according to BMI. Women with unlikely weights (≤ 37 kg or ≥ 187 kg), or who do not fall into one of the identified weight groups were excluded (n = 96,411 excluded). Of the remaining births, one birth per mother was randomly selected (n = 53,865 excluded). Births to mothers with pre-existing type I or type II diabetes (n = 1218) as well as a pre-existing mood/anxiety disorder (n = 13,128) were excluded, since both pre-existing conditions are predictors for postpartum depression [32–34]. Therefore, the final weight group cohorts are N = 17,975 for the lean group, and N = 9908 for the with obesity group (Fig. 1).

### Primary outcome

A woman was considered to have PPD if in the 12 months after giving birth she had: (i) one or more hospitalizations with a diagnosis of depressive disorder, affective psychoses, neurotic depression, adjustment reaction, anxiety disorder, anxiety states, phobic disorders, or obsessive–compulsive disorders according to International Classification of Disease, 9^th^ revision, Clinical Modification (ICD-9-CM) codes: 296.2–296.8, 300, 300.0, 300.2, 300.3, 300.4, 309, 311, or ICD 10^th^ revision Canada (ICD-10-CA) codes: F31, F32, F32.0, F33, F34.1, F38.0, F38.1, F40, F41, F41.0, F41.1, F41.2, F41.3, F41.8, F41.9, F42, F43.1, F43.2, F43.8, F44, F45.0, F451, F452, F48, F53.0, F68.0, F93.0, F99; (ii) one or more physician visits with a diagnosis of depressive disorder, affective psychoses, or adjustment reaction according to ICD-9-CM codes 296, 309 or 311; (iii) a prescription for an antidepressant or mood stabilizer based on Anatomical Therapeutic Chemical (ATC) codes N03AB02, N03AB52, N03AF01, N05AN01, N06A; (iv) one or more physician visits with a diagnosis for anxiety disorders (ICD-9-CM code 300) as well as one or more prescriptions for an antidepressant or mood stabilizer (ATC codes N03AB02, N03AB52, N03AF01, N05AN01, N06A); and (v) two or more physician visits with a diagnosis for anxiety disorders (ICD-9-CM code 300). This definition has been used in previous studies at MCHP (http://mchp-appserv.cpe.umanitoba.ca/viewDefinition.php?definitionID=104493).

### Covariates

The following information was extracted for all women: maternal age at pregnancy start date (continuous and 5-year age groups), area-level income (urban and rural quintiles), insulin treatment in pregnancy (yes/no; based on a dispensed prescription with ATC code A10A filled in the 168 days following pregnancy start date and before the child’s birth date), pre-existing diabetes (yes/no), and pre-existing mood/anxiety disorders (yes/no).

Pre-existing diabetes and pre-existing mood/anxiety disorders were determined before limiting data to one random birth per mother so that if either of these conditions were present on any pregnancy they could be “carried forward” to all subsequent pregnancies for that mother. Thus, pre-existing diabetes was defined as: (i) having any type of diabetes in the two years prior to the pregnancy start date, or in the 168 days (24-weeks) following the pregnancy start date or (ii) having pre-existing diabetes diagnosed in a previous pregnancy. Pre-existing diabetes was identified using an existing algorithm at MCHP [35] (see Additional file 2 for full definition). Pre-existing mood/anxiety disorder was defined as: (i) having a mood and/or anxiety disorder diagnosed in the 5-years prior to pregnancy start or (ii) having a pre-existing mood and/or anxiety disorder diagnosed in a previous pregnancy. A mood/anxiety disorder was defined using an existing algorithm at MCHP [35] (see Additional file 2 for full definition).

### Statistical analysis

All data management, programming and analyses were performed on MCHP’s secure servers, using SAS® version 9.4 software. The percentage (%) and adjusted rate of PPD, as well as 95% confidence intervals (CIs), were estimated using Poisson distributions containing a log link function and the logarithm of the number of mothers as the offset in the models. Models were adjusted for maternal age group (at pregnancy start date) and area-level income (at delivery). While it would have been ideal to adjust for other factors associated with both insulin treatment and obesity, the sample size did not allow for additional adjustment. The analyses were stratified by insulin treatment (yes/no) in pregnancy. Significance is represented by a *p* value being < 0.05 (two-tailed).

## Results

Demographic characteristics of the mothers in this study are provided in Tables 1 and 2.

**Table 1:** Demographic characteristics of mothers at the time of delivery by weight group

Variable	Levels	Lean		Obesity	
		N	%	N	%
Weight group		17,975		9908	
Maternal age groups at the start of Pregnancy	15–19	2678	14.90	703	7.10
	20–25	4369	24.31	2480	25.03
	26–30	5039	28.03	3088	31.17
	30–35	4144	23.05	2537	25.61
	36 +	1745	9.71	1100	11.10
Mean maternal age (Mean, Standard Deviation)		27.19	6.37	28.32	5.73
Income quintiles	Urban Lowest (U1)	2363	13.15	1160	11.71
	U2	2422	13.47	1225	12.36
	U3	2274	12.65	1071	10.81
	U4	2364	13.15	976	9.85
	Urban Highest (U5)	2401	13.36	671	6.77
	Rural Lowest (R1)	1475	8.21	1415	14.28
	R2	1149	6.39	1023	10.32
	R3	982	5.46	679	6.85
	R4	1078	6.00	827	8.35
	Rural Highest (R5)	1349	7.50	828	8.36
	Not Found	118	0.66	33	0.33
Insulin received during pregnancy		42	0.23	238	2.40

Mothers with pre-existing type I or type II *diabetes mellitus* or with pre-existing mood/anxiety disorders were excluded. The number (N) and percentages (%) are shown unless otherwise specified

**Table 2:** Percentage of postpartum psychological distress within the first year following delivery by weight group

Weight group	Postpartum psychological distress			
	n	%	95% CI	
			Lower	Upper
Lean (N = 17,975)	1246	6.93	6.56	7.33
Obesity (N = 9908)	848	8.56	8.00	9.15

Mothers with pre-existing type I or type II *diabetes mellitus* or with pre-existing mood/anxiety disorders were excluded. The number (N), percentage (%) and 95% confidence intervals (CI) are shown

The mean age at the time of delivery was similar between mothers with and without obesity (28.32 vs. 27.19 years respectively) (Table 1). The income gradient shows that more mothers had lower income (lower three urban and rural income quintiles), especially among those with obesity (Table 1). In the lean group, < 1.0% of mothers were treated with insulin during pregnancy while in the obesity group, 2.40% of mothers were treated with insulin (Table 1). The risk of PPD was higher in the obesity group (8.56%; 95% CI 8.00–9.15) than in the lean group (6.93%; 95% CI 6.56–7.33) (Table 2).

**Fig. 2:**
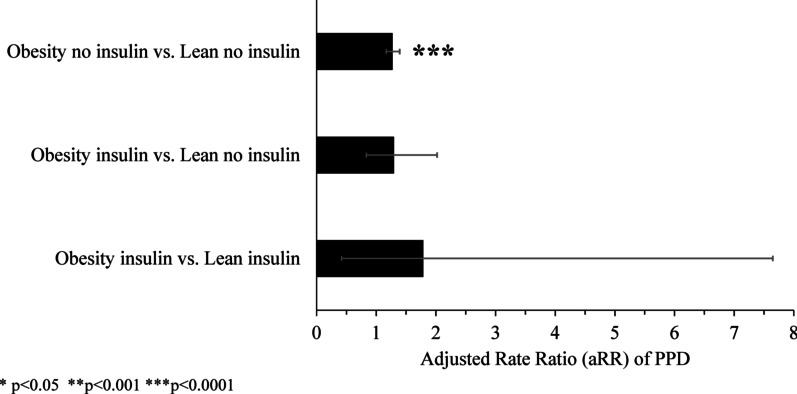
Direct comparisons of the rates of postpartum psychological distress (PPD) within one year following delivery between weight groups and insulin treatment. Adjusted rate ratios (aRR) and 95% confidence intervals are shown

The percentage (%) and adjusted rate ratios (aRRs) of PPD among mothers within the first year following delivery with and without insulin treatment during pregnancy are shown in Table 3. The percentage of mothers with obesity and PPD decreased with insulin treatment however, this decrease did not reach significance (aRR 1.02, 95% CI 0.65–1.59, *p* = 0.936). Therefore, we performed a secondary exploratory analysis to investigate how insulin treatment in pregnancy affects the risk of PPD in women with obesity relative to women without obesity (Fig. 2 and Table 4). This analysis reflects our previous study results, where we found women with obesity who received insulin to treat GDM had increased PL levels, which met and exceeded the levels detected in the otherwise healthy lean group of women [21]. Thus, we hypothesized that insulin treatment will reduce the risk of PPD among women with obesity to the level of risk seen among women without obesity. When no insulin treatment was given during pregnancy, the obesity group had a significantly higher risk of PPD than the lean group (aRR 1.27; 95% CI 1.17–1.39; *p* < 0.0001). Interestingly, when mothers with obesity treated with insulin were compared to mothers in the lean group not treated with insulin, no significant difference was seen in rates of PPD (aRR 1.30; 95% CI 0.83–2.02; *p* = 0.248) (Fig. 2 and Table 4). This is suggestive that insulin changes the relationship between obesity and PPD.

**Table 3:** Percentage and rate of postpartum psychological distress among mothers within the first year following delivery by weight group and insulin treatment during pregnancy

Weight group	Received insulin during pregnancy	Postpartum psychological distress							
		n	%	95% CI		Adjusted rate ratio (aRR)	95% CI		*p* value
				Lower	Upper		Lower	Upper	
Lean	No (N = 17,933)	1244	6.94	6.56	7.33	0.73	0.18	2.91	0.651
	Yes (N = 42)	s	s	s	s				
Obesity	No (N = 9670)	828	8.56	8.00	9.17	1.02	0.65	1.59	0.936
	Yes (N = 238)	20	8.40	5.42	13.03				

Mothers with pre-existing type I or type II *diabetes mellitus* or with pre-existing mood/anxiety disorders were excluded. The number (N), percent (%), adjusted rate ratios (aRR) and 95% confidence intervals (CI) are shown. Rate ratios were adjusted for maternal age group (at pregnancy start date) and area-level income (at delivery)

“s” = data suppressed due to small numbers

**Table 4:** Direct comparisons of the rate of postpartum psychological distress among mothers within the first year following delivery by insulin and weight groups

Comparison groups	Postpartum psychological distress			
	Adjusted rate ratio (aRR)	95% CI		*p* value
		Lower	Upper	
Obesity insulin vs. Lean insulin	1.79	0.42	7.65	0.434
Obesity insulin vs. Lean no insulin	1.30	0.83	2.02	0.248
Obesity no insulin vs. Lean no insulin	1.27	1.17	1.39	< 0.0001

Adjusted rate ratios (aRR) and 95% confidence intervals (CI) are show. Mothers with pre-existing type I or type II *diabetes mellitus* or with pre-existing mood/anxiety disorders were excluded. Rate ratios were adjusted for maternal age group (at pregnancy start date) and area-level income (at delivery). Rate ratios were calculated by dividing the Obesity group (± insulin) PPD rate by the Lean group (± insulin) PPD rate

## Discussion

In this study, our first objective was to identify the risk of PPD among women with and without obesity. We found that maternal obesity is associated with a significant increase in the rate of PPD within one year after delivery in Manitoba, Canada. Our second objective was to observe the effect of insulin treatment during pregnancy on the risk of PPD. Interestingly, when women with obesity and insulin treatment were compared to the lean group with no insulin treatment, significance was lost, providing support to the hypothesis that insulin changes the relationship between obesity and PPD. Therefore, our results support a relationship between obesity and peripartum depression, where the potential benefit of insulin treatment may reflect positive effects on both glycemic control and on lactogenic hormone levels [21].

Furthermore, decreased human placental lactogen (PL) production has been identified as a marker of clinical antenatal depression [19], and obesity is also associated with reduced human PL levels and peripartum depression [5, 20, 21, 25], highlighting the importance of lactogenic (hormonal) signaling in the brain. Several studies in mice provide a link between decreased lactogen levels in the brain and impaired maternal behaviors [36, 37], further highlighting the importance of lactogenic signaling in the brain and its role in mediating behaviors. Abnormalities in gene expression of various neurotransmitters, neuropeptides, neutrophins and hormones undoubtedly underlie the neurobiology of mental disorders, particularly at the molecular level [38]. While human PL is known to stimulate insulin production as a result of increasing pancreatic β-cell mass [39], the mechanism for positive regulation of human PL synthesis by insulin is not yet reported, nor is the mechanism with which it mediates its positive effects in the brain. However, the rescue of human PL gene expression seen in women with obesity that develop GDM and are treated with insulin [21], is consistent with rescue of both lactogenic signaling via the prolactin receptor and related negative effects in the maternal brain.

No study is without limitation. In our primary analysis we did not observe a significant difference in the direct comparisons of PPD in women with obesity who received insulin relative to women with obesity without treatment (Table 3). However, it is likely that our inability to detect a significant difference was due to the low incidence of both insulin treatment in pregnancy and PPD after pregnancy resulting in small cohort numbers. This resulted in a rate that may change significantly based on the inclusion or exclusion of a small number of women.

It is also important to note that GDM itself is suggested to be a risk factor for peripartum depressive symptoms [24, 27, 40–42]. A recent systematic review of relevant epidemiological studies identified 18 studies with a sample size of 2,370,958 for review [27]. These studies were subjected to meta-analysis where the authors found that GDM significantly increases the risk of postpartum depression (relative risk of 1.59; 95% CI 1.22–2.07, *p* = 0.001) [27]. A study effectuated in Brazil on a cohort of female participants who were pregnant and were diagnosed with GDM (N = 820) aimed to assess the frequency and severity of depressive symptoms (according to the Edinburgh Postnatal Depression Scale) and their relationship to clinical and sociodemographic characteristics [24]. The author’s found that 47% of female participants were obese before their pregnancies, 31% showed depressive symptoms and ~ 10% showed severe symptoms. However, only 12% of the women evaluated were using insulin to treat their GDM [24]. In addition, pre-pregnancy BMI and insulin use during pregnancy were not associated with depressive symptoms in any level after prevalence ratios were adjusted [24]. Interestingly, another study effectuated in the United States that looked at postpartum women whose most recent pregnancy was complicated by GDM (N = 71) reported no significant association between depressive symptoms and insulin use during pregnancy [40]. This further supports our findings that insulin evokes a positive effect on mental health outcomes.

Our hypothesis was formulated on the basis of our previous findings in mouse [20] and in human placental [21] studies, where the latter only included pregnant women with a BMI of 35 or higher [21]. In the current study, our weight range was determined based on all three BMI classifications. This was done in part to prevent small cohort numbers, but the sensitivity of the effect of insulin treatment during pregnancy may have been lost, resulting in non-significant results. Studies show that the effects of BMI on health and mortality is heterogenous and becomes increasingly worse with increasing BMI class [43–46].

Another limitation to the study was in inability to use BMI to identify our cohorts of pregnant women. BMI values are not identifiable in the administrative data unless self-reported survey data is used. However, surveys only sample a portion of the population and there is considerable under- and over-reporting of BMI values [47]. The use of BMI to classify individuals and its utility as a measure of health has been consistently contested in the literature [48, 49]. A recent Danish study looked at the association between weight and depressive symptoms in a group of women followed for up to 16 years after their first birth [50]. They also studied if waist circumference (WC) adjusted for BMI (WC_BMI_) 7 years after childbirth (as an indicator for abdominal adiposity) was associated with depressive symptoms. The authors found that women with a larger waist circumference than predicted by their BMI reported more depressive symptoms than women with a WC in accordance with their BMI [50]. In our study, we estimated a 9% misclassification rate among the women with obesity. Therefore, it is possible that women with overweight status may have been included, biasing our results towards the null. In addition, the maternal weight identified in our data was a recorded weight during a prenatal care/physician visit during early or mid-pregnancy and is not representative of pre-conception maternal weight further contributing to misclassification.

## Conclusion

Maternal obesity is associated with a higher incidence of PPD within one year after delivery in Manitoba, Canada when compared to lean women. Without insulin treatment in pregnancy, women with obesity had a significantly higher risk of PPD when compared to lean women. While the direct comparison between women with obesity treated with insulin and those without insulin treatment did not attain significance, mothers with obesity who received insulin in pregnancy have similar rates of PPD to lean mothers who did not receive insulin. This suggests an added benefit to maintaining sufficient insulin levels in pregnancy beyond the requirement to regulate blood sugar among women with obesity, and may be a preventative measure against PPD in order to better support maternal care of offspring.

With additional data years, the study can be expanded to allow assessment of the type of glucose management (e.g., insulin, metformin or euglycemic diet management only) on the risk of PPD in mothers with GDM and obesity. In terms of mechanism and effects on brain physiology and maternal behavior, the effect of insulin treatment in pregnant females with obesity can also be modelled in a partially humanized mouse containing (and expressing) the human PL gene family. This model has already demonstrated the negative effect of an acute high fat diet-induced obesity on human PL levels during pregnancy [20]. More specifically, this mouse model may allow investigation of whether the beneficial effect of insulin is through glycemic control only (e.g., maintaining energy homeostasis) or whether there is a role for indirect interactions with other hormones (e.g., hypothalamic–pituitary–adrenal axis etc.). Our previous human study [21] can also be expanded to include multiple maternal serum sampling time points as well as placental tissue sampling at term from four groups of pregnant women in order to observe the effect of insulin and euglycemic diet on human PL production. These women can also be monitored for up to one year after birth in order to screen for postpartum depression. Thus, the current study as well as these future studies would provide more evidence to support PL/CS hormone levels as a potential marker of peripartum depression and insulin treatment as a possible preventative measure.

## Supplementary Information

**Additional file 1**. Approach, calculation and validation of weight cut-off ranges for the “with obesity” and “without obesity” groups. **A**. Flowchart representing a summary of how weight cut-off ranges were calculated using average female height and weight, followed by reverse BMI calculation (image created by first author using BioRender.com). **B**. Mathematics and subsequent details involved in the calculation of weight cut-off ranges. **C**. Validation of weight ranges.

**Additional file 2**. “Diabetes” and “Mood and Anxiety Disorder” Definitions and List of Codes. The detailed operational definitions and SAS® program code for “diabetes” and “Mood and Anxiety Disorder”, developed from administrative data.

## Data Availability

The data was provided under specific data sharing agreements only for approved secondary use at Manitoba Centre for Health Policy (MCHP). The original source data is not owned by the researchers or MCHP and as such cannot be provided to a public repository. The data that supports the findings of this study may be provided from the corresponding author upon reasonable request. Where necessary, source data specific to this article or project may be reviewed at MCHP with the consent of the original data providers, along with the required privacy and ethical review bodies.

## Abbreviations

aRR: Adjusted rate ratio
ATC: Anatomical Therapeutic Chemical
BMI: Body mass index
CDC: Centers for Disease Control and Prevention
CI: Confidence interval
CS: Chorionic somatomammotropin
DERCA: Diabetes Education Resource for Children and Adolescents
DPIN: Drug Program Information Network
DSM: Diagnostic Services of Manitoba
GDM: Gestational *diabetes mellitus* (GDM)
ICD-9-CM: International Classification of Disease, 9th revision, Clinical Modification
ICD-10-CA: International Classification of Disease, 10th revision, Canada
MCHP: Manitoba Centre for Health Policy
MMSSP: Manitoba Maternal Serum Screening Program
N: Number
nblink: Hospital Newborn to Mother Link Registry
PHIN: Personal Health Identification Number
PL: Placental lactogen
PPD: Postpartum psychological distress
PRL: Prolactin
RNA: Ribonucleic acid
WC: Waist circumference
WC_BMI_: Waist circumference adjusted for BMI

## References

[1] Statistics Canada. Table 13-10-0096-01 Health characteristics, annual estimates. 2019. https://www150.statcan.gc.ca/t1/tbl1/en/tv.action?pid=1310009601. Accessed 27 Nov 2019.

[2] Vinturache A, Moledina N, McDonald S, Slater D, Tough S (2014). Pre-pregnancy Body Mass Index (BMI) and delivery outcomes in a Canadian population. BMC Pregnancy Childbirth.

[3] Wei YM, Yang HX, Zhu WW, Liu XY, Meng WY, Wang YQ (2015). Risk of adverse pregnancy outcomes stratified for pre-pregnancy body mass index. J Matern Neonatal Med.

[4] Kulie T, Slattengren A, Redmer J, Counts H, Eglash A, Schrager S (2011). Obesity and women’s health: an evidence-based review. J Am Board Fam Med.

[5] Steinig J, Nagl M, Linde K, Zietlow G, Kersting A (2017). Antenatal and postnatal depression in women with obesity: a systematic review. Arch Womens Ment Health.

[6] Cunningham SD, Mokshagundam S, Chai H, Lewis JB, Levine J, Tobin JN (2018). Postpartum depressive symptoms: gestational weight gain as a risk factor for adolescents who are overweight or obese. J Midwifery Women’s Heal.

[7] LaCoursiere DY, Barrett-Connor E, O’Hara MW, Hutton A, Varner MW (2010). The association between prepregnancy obesity and screening positive for postpartum depression. BJOG.

[8] Schofield Z, Kapoor D (2019). Pre-existing mental health disorders and pregnancy. Obstet Gynaecol Reprod Med.

[9] Binder EB, Newport DJ, Zach EB, Smith AK, Deveau TC, Altshuler LL (2010). A serotonin transporter gene polymorphism predicts peripartum depressive symptoms in an at-risk psychiatric cohort. J Psychiatr Res.

[10] Galea LAM, Frokjaer VG (2019). Perinatal depression: embracing variability toward better treatment and outcomes. Neuron.

[11] Guille C, Newman R, Fryml LD, Lifton CK, Epperson CN (2013). Management of postpartum depression. J Midwifery Womens Health.

[12] Gude NM, Roberts CT, Kalionis B, King RG (2004). Growth and function of the normal human placenta. Thromb Res.

[13] Napso T, Yong HEJ, Lopez-Tello J, Sferruzzi-Perri AN (2018). The role of placental hormones in mediating maternal adaptations to support pregnancy and lactation. Front Physiol.

[14] Bridges RS (2015). Neuroendocrine regulation of maternal behavior. Front Neuroendocrinol.

[15] Freemark M (2010). Placental hormones and the control of fetal growth. J Clin Endocrinol Metab.

[16] Sonagra AD, Biradar SM, Murthy J (2014). Normal pregnancy—a state of insulin resistance. J Clin Diagnostic Res.

[17] Kampmann U, Knorr S, Fuglsang J, Ovesen P (2019). Determinants of maternal insulin resistance during pregnancy: an updated overview. J Diabetes Res.

[18] Sumption LA, Garay SM, John RM (2020). Low serum placental lactogen at term is associated with postnatal symptoms of depression and anxiety in women delivering female infants. Psychoneuroendocrinology.

[19] Janssen AB, Capron LE, O’Donnell K, Tunster SJ, Ramchandani PG, Heazell AEP (2016). Maternal prenatal depression is associated with decreased placental expression of the imprinted gene PEG3. Psychol Med.

[20] Vakili H, Jin Y, Menticoglou S, Cattini PA (2013). CCAAT-enhancer-binding protein β (C/EBPβ) and downstream human placental growth hormone genes are targets for dysregulation in pregnancies complicated by maternal obesity. J Biol Chem.

[21] Jin Y, Vakili H, Liu SY, Menticoglou S, Bock ME, Cattini PA (2018). Chromosomal architecture and placental expression of the human growth hormone gene family are targeted by pre-pregnancy maternal obesity. Am J Physiol Endocrinol Metab.

[22] Muralimanoharan S, Maloyan A, Myatt L (2016). Mitochondrial function and glucose metabolism in the placenta with gestational diabetes mellitus: role of miR-143. Clin Sci (Lond).

[23] Sibiak R, Jankowski M, Gutaj P, Mozdziak P, Kempisty B, Wender-Ożegowska E (2020). Placental lactogen as a marker of maternal obesity, diabetes, and fetal growth abnormalities: current knowledge and clinical perspectives. J Clin Med.

[24] Damé P, Cherubini K, Goveia P, Pena G, Galliano L, Façanha C (2017). Depressive symptoms in women with gestational diabetes mellitus: the LINDA-Brazil Study. J Diabetes Res..

[25] Molyneaux E, Poston L, Ashurst-Williams S, Howard LM (2014). Obesity and mental disorders during pregnancy and postpartum: a systematic review and meta-analysis. Obstet Gynecol.

[26] Rogan SC, Payne JL, Meltzer-Brody S, Nicholson W, Baptiste-Roberts K (2014). Relationship between depressive mood and maternal obesity: implications for postpartum depression. Obesity during pregnancy in clinical practice.

[27] Azami M, Badfar G, Soleymani A, Rahmati S (2019). The association between gestational diabetes and postpartum depression: a systematic review and meta-analysis. Diabetes Res Clin Pract.

[28] Roos LL, Brownell M, Lix L, Roos NP, Walld R, MacWilliam L (2008). From health research to social research: privacy, methods, approaches. Soc Sci Med.

[29] Jutte DP, Roos LL, Brownell MD (2011). Administrative record linkage as a tool for public health research. Annu Rev Public Health.

[30] Fryar CD, Kruszon-Moran D, Gu Q, Ogden C. Mean Body Weight, Height, Waist Circumference, and Body Mass Index Among Adults: United States, 1999–2000 Through 2015–2016. 2018. https://www.cdc.gov/nchs/data/nhsr/nhsr122-508.pdf. Accessed 27 Nov 2019. 30707668

[31] Centers for Disease Control and Prevention. 2017 Data Release. National Health Interview Survey. 2017. https://www.cdc.gov/nchs/nhis/nhis_2017_data_release.htm. Accessed 27 Nov 2019.

[32] Ghaedrahmati M, Kazemi A, Kheirabadi G, Ebrahimi A, Bahrami M (2017). Postpartum depression risk factors: a narrative review. J Educ Health Promot.

[33] Gelaye B, Rondon MB, Araya R, Williams MA (2016). Epidemiology of maternal depression, risk factors, and child outcomes in low-income and middle-income countries. Lancet Psychiatry.

[34] Silverman ME, Reichenberg A, Savitz DA, Cnattingius S, Lichtenstein P, Hultman CM (2017). The risk factors for postpartum depression:a population-based study. Depress Anxiety.

[35] Fransoo R, Mahar A, The Need to Know Team, Anderson A, Prior H, Koseva I, et al. The 2019 RHA Indicators Atlas. Winnipeg, Manitoba; 2019. http://mchp-appserv.cpe.umanitoba.ca/reference//RHA_Report_web.pdf. Accessed 27 Nov 2019.

[36] Shingo T, Gregg C, Enwere E, Fujikawa H, Hassam R, Geary C (2003). Pregnancy-stimulated neurogenesis in the adult female forebrain mediated by prolactin. Science (80-).

[37] Lucas BK, Ormandy CJ, Binart N, Bridges RS, Kelly PA (1998). Null mutation of the prolactin receptor gene produces a defect in maternal behavior. Endocrinology.

[38] Pawluski JL, Lonstein JS, Fleming AS (2017). The neurobiology of postpartum anxiety and depression. Trends Neurosci.

[39] Newbern D, Freemark M (2011). Placental hormones and the control of maternal metabolism and fetal growth. Curr Opin Endocrinol Diabetes Obes.

[40] Nicklas JM, Miller LJ, Zera CA, Davis RB, Levkoff SE, Seely EW (2013). Factors associated with depressive symptoms in the early postpartum period among women with recent gestational diabetes mellitus. Matern Child Health J.

[41] Putnick DL, Sundaram R, Bell EM, Ghassabian A, Goldstein RB, Robinson SL (2020). Trajectories of maternal postpartum depressive symptoms. Pediatrics.

[42] Hinkle SN, Buck Louis GM, Rawal S, Zhu Y, Albert PS, Zhang C (2016). A longitudinal study of depression and gestational diabetes in pregnancy and the postpartum period. Diabetologia.

[43] Nyberg ST, Batty GD, Pentti J, Virtanen M, Alfredsson L, Fransson EI (2018). Obesity and loss of disease-free years owing to major non-communicable diseases: a multicohort study. Lancet Public Heal.

[44] Edqvist J, Rawshani A, Adiels M, Björck L, Lind M, Svensson A-M (2018). BMI and mortality in patients with New-Onset Type 2 diabetes: a comparison with age- and sex-matched control subjects from the general population. Diabetes Care.

[45] Kim SS, Zhu Y, Grantz KL, Hinkle SN, Chen Z, Wallace ME (2016). Obstetric and neonatal risks among obese women without chronic disease. Obstet Gynecol.

[46] Scott-Pillai R, Spence D, Cardwell CR, Hunter A, Holmes VA (2013). The impact of body mass index on maternal and neonatal outcomes: a retrospective study in a UK obstetric population, 2004–2011. BJOG.

[47] Shields M, Connor Gorber S, Tremblay MS (2008). Estimates of obesity based on self-report versus direct measures. Heal Rep.

[48] Gutin I (2018). In BMI we trust: reframing the body mass index as a measure of health. Soc Theory Health.

[49] Ortega FB, Sui X, Lavie CJ, Blair SN (2016). Body mass index, the most widely used but also widely criticized index: would a criterion standard measure of total body fat be a better predictor of cardiovascular disease mortality?. Mayo Clin Proc.

[50] Bliddal M, Pottegård A, Kirkegaard H, Olsen J, Sørensen TIA, Nohr EA (2016). Depressive symptoms in women’s midlife in relation to their body weight before, during and after childbearing years. Obes Sci Pract.

