# Supplementary material for: A potential role for insulin treatment during pregnancy in reducing postpartum psychological distress in maternal obesity: an administrative population health study
Source: BMC Womens Health. 2021 Mar 20;21:117. doi: 10.1186/s12905-021-01261-0 (PMC7981977; doi:10.1186/s12905-021-01261-0)
Supplement: Supplementary file 1 — Additional file 1. Approach, calculation and validation of weight cut-off ranges for the “with obesity” and “without obesity” groups. A. Flowchart representing a summary of how weight cut-off ranges were calculated using average female height and weight, followed by reverse BMI calculation (image created by first author using BioRender.com). B. Mathematics and subsequent details involved in the calculation of weight cut-off ranges. C. Validation of weight ranges. [file 12905_2021_1261_MOESM1_ESM.docx]

**Additional File 1: Approach, calculation and validation of weight cut-off ranges for the “with obesity” and “without obesity” groups.**

1. **Flowchart representing a summary of how we calculated weight cut-off ranges using average female height and weight, followed by reverse BMI calculation.**

**
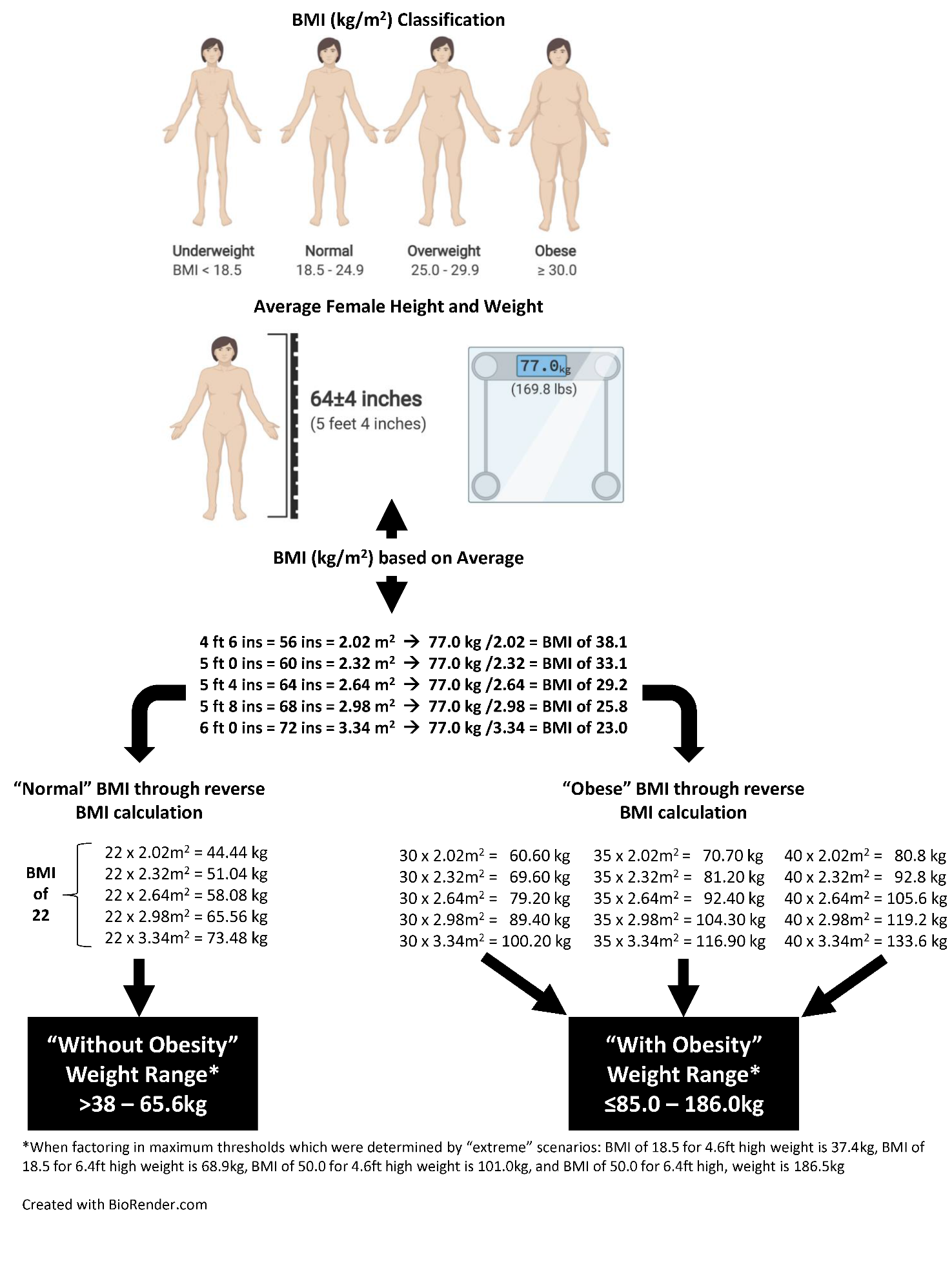
**

1. **Mathematics and details involved in the calculation of weight cut-off ranges**

**Average female height = 63.6 inches rounded to 64 inches plus or minus 4 inches**

**Average weight = 169.8 pounds (77.0kg)**

**According to CDC report**^[[1]](#footnote-1)^ **= 2013-2014 data (crude value in women 20 years and over)**

| **BMI (kg/m^2)^** | **Classification^[[2]](#footnote-2)^** |  |
| --- | --- | --- |
| **< 18.5** | **Underweight** |  |
| **18.5 – 24.9** | **Normal** |  |
| **25.0 – 29.9** | **Overweight** | **BMI Formula: weight (kg) / [height (m)]^2^** |
| **30.0 – 34.9** | **Obese, Class I** |  |
| **35.0 – 39.9** | **Obese, Class II** |  |
| **>40.0 kg/m^2^** | **Obese, Class III** |  |

**Average BMI based on height (64±4 inches) with average weight of 77.0kg:**

**4 ft 6 ins = 56 ins = 1.4224 m = 2.02 m^2^ 77.0 kg (169.8 lbs) /2.02 = BMI = 38.1**

**5 ft 0 ins = 60 ins = 1.5240 m = 2.32 m^2^ 77.0 kg (169.8 lbs) /2.32 = BMI = 33.1**

**5 ft 4 ins = 64 ins = 1.6256 m = 2.64 m^2^ 77.0 kg (169.8 lbs) /2.64 = BMI = 29.2**

**5 ft 8 ins = 68 ins = 1.7272 m = 2.98 m^2^ 77.0 kg (169.8 lbs) /2.98 = BMI = 25.8**

**6 ft 0 ins = 72 ins = 1.8288 m = 3.34 m^2^ 77.0 kg (169.8 lbs) /3.34 = BMI = 23.0**

**1. Determining a “normal” BMI through reverse BMI calculation using the average female height of 64 inches (1.6256m) and an average BMI of 22 (normal classification):**

**4 ft 6 ins = 56 ins = 1.4224 m = 2.02 m2 BMI = 22 = 22 x 2.02 = 44.44 kg**

**5 ft 0 ins = 60 ins = 1.5240 m = 2.32 m2 BMI = 22 = 22 x 2.32 = 51.04 kg**

**5 ft 4 ins = 64 ins = 1.6256 m = 2.64 m2 BMI = 22 = 22 x 2.64 = 58.08 kg**

**5 ft 8 ins = 68 ins = 1.7272 m = 2.98 m2 BMI = 22 = 22 x 2.98 = 65.56 kg**

**6 ft 0 ins = 72 ins = 1.8288 m = 3.34 m2 BMI = 22 = 22 x 3.34 = 73.48 kg**

**Therefore, for a height of 64±4 inches and a BMI = 22, estimated “without obesity” weight range = 51.0-65.6 kg**

**2. Determining an “obese” BMI through reverse BMI calculation using the average female height of 64 inches and an average BMI of 30, 35 and 40 (with obesity):**

**4 ft 6 ins = 56 ins = 1.4224 m = 2.02 m2 BMI = 30 = 30 x 2.02 = 60.60 kg**

**5 ft 0 ins = 60 ins = 1.5240 m = 2.32 m2 BMI = 30 = 30 x 2.32 = 69.60 kg**

**5 ft 4 ins = 64 ins = 1.6256 m = 2.64 m2 BMI = 30 = 30 x 2.64 = 79.20 kg**

**5 ft 8 ins = 68 ins = 1.7272 m = 2.98 m2 BMI = 30 = 30 x 2.98 = 89.40 kg**

**6 ft 0 ins = 72 ins = 1.8288 m = 3.34 m2 BMI = 30 = 30 x 3.34 = 100.20 kg**

**4 ft 6 ins = 56 ins = 1.4224 m = 2.02 m2 BMI = 35 = 35 x 2.02 = 70.70 kg**

**5 ft 0 ins = 60 ins = 1.5240 m = 2.32 m2 BMI = 35 = 35 x 2.32 = 81.20 kg**

**5 ft 4 ins = 64 ins = 1.6256 m = 2.64 m2 BMI = 35 = 35 x 2.64 = 92.40 kg**

**5 ft 8 ins = 68 ins = 1.7272 m = 2.98 m2 BMI = 35 = 35 x 2.98 = 104.30 kg**

**6 ft 0 ins = 72 ins = 1.8288 m = 3.34 m2 BMI = 35 = 35 x 3.34 = 116.90 kg**

**4 ft 6 ins = 56 ins = 1.4224 m = 2.02 m2 BMI = 40 = 40 x 2.02 = 80.8 kg**

**5 ft 0 ins = 60 ins = 1.5240 m = 2.32 m2 BMI = 40 = 40 x 2.32 = 92.8 kg**

**5 ft 4 ins = 64 ins = 1.6256 m = 2.64 m2 BMI = 40 = 40 x 2.64 = 105.6 kg**

**5 ft 8 ins = 68 ins = 1.7272 m = 2.98 m2 BMI = 40 = 40 x 2.98 = 119.2 kg**

**6 ft 0 ins = 72 ins = 1.8288 m = 3.34 m2 BMI = 40 = 40 x 3.34 = 133.6 kg**

**Therefore, for height of 64±4 ins and a BMI of 30 up to 40, estimated average weight range = 69.6 – 119.2 kg**

**Maximum thresholds were determined by “extreme” scenarios:**

- **A BMI of 18.5 for 4.6ft high, weight is 37.4kg**
- **A BMI of 18.5 for 6.4ft high, weight is 68.9kg**
- **A BMI of 50.0 for 4.6ft high, weight is 101.0kg**
- **A BMI of 50.0 for 6.4ft high, weight is 186.5kg**

**Taken together, final weight group cut-offs:**

**Lean (without obesity) =** >38-65.6kg **Obesity (with obesity) =** ≤85.0-186kg, with ≤85.0 being determined during validation.

Women with unlikely weights (≤37kg or ≥187kg) or who do not fall into one of the weight groups were therefore excluded.

1. **V****alidation of weight ranges to a previous study:**

**Analysis of available pilot data from our previous study (*Jin et al., 2018. Am J Physiol Endocrinol Metab 315: E435–E445* and *Vakili et al. 2013. J Biol Chem; 288(31): 22849–22861*), also supports identifying no obesity and with obesity cohorts of pregnant women based on pre-pregnancy weights of < 65.6 kg versus > 85.0 kg, respectively. We attempted to validate these “cutoffs” by using the recorded height, weight and BMI values available from the women in the study which compared lean (non-obese) versus obese women.**

**Pre-pregnancy weight cutoff <65.6 kg**

- **39 = women with a pre-pregnancy BMI of <25 kg/m^2^ in *Jin et al. 2018 & Vakili et al. 2013***
- **35 of these women had a pre-pregnancy weight of < 65.6 kg**
- **35 / 39 = 0.897*100 = 90% capture rate**

**Pre-pregnancy weight cutoff >85.0 kg**

- **23 = women with a pre-pregnancy BMI of >30 kg/m^2^ in *Jin et al. 2018 & Vakili et al. 2013***
- **23 of these women had a pre-pregnancy weight > 85.0 kg**
- **23/23 = 0.1*100 = 100% capture rate**

**Thus, this sample analysis above supports designation of no obesity and with obesity cohorts of pregnant women based on weights of < 65.6 kg versus > 85 kg, respectively.**

1. Table 1 and Table 3 in: <https://www.cdc.gov/nchs/data/nhsr/nhsr122-508.pdf> [↑](#footnote-ref-1)
2. Source: Health Canada. Canadian Guidelines for Body Weight Classification in Adults. Ottawa: Minister of Public Works and Government Services Canada; 2003. [↑](#footnote-ref-2)
