# Supplementary material for: A potential role for insulin treatment during pregnancy in reducing postpartum psychological distress in maternal obesity: an administrative population health study
Source: BMC Womens Health. 2021 Mar 20;21:117. doi: 10.1186/s12905-021-01261-0 (PMC7981977; doi:10.1186/s12905-021-01261-0)
Supplement: Supplementary file 2 — Additional file 2. “Diabetes” and “Mood and Anxiety Disorder” Definitions and List of Codes. The detailed operational definitions and SAS® program code for “diabetes” and “Mood and Anxiety Disorder”, developed from administrative data. [file 12905_2021_1261_MOESM2_ESM.docx]

**Additional File 2:** **“Diabetes” and “Mood and Anxiety Disorder” Definitions and List of Codes**

**Diabetes**

Diabetes was defined by one of the following conditions:

- one or more hospitalizations with a diagnosis of diabetes: ICD–9–CM code 250, or ICD–10–CA codes E10–E14;
- two or more physician visits in two years with a diagnosis of diabetes (ICD–9–CM code 250);
- one or more prescriptions for medications to treat diabetes (ATC code A10, specific drugs that were included are listed below) UNLESS the prescriptions are all for metformin (ATC code A10BA) without any other diabetes prescriptions, no diagnoses for diabetes from a hospital or physician visit, no high HgA1c test, and no record in the Diabetes Education Resource for Children and Adolescents database;
- one or more glycohemoglobin (HgA1c) tests with a result ≥ 6.5;
- identified as having diabetes in the Diabetes Education Resource for Children and Adolescents database.

List of drug Anatomic Therapeutic Chemical (ATC) codes and generic drug names used to treat diabetes:

| - A10A - Insulins and Analogues - A10BA02 - Metformin - A10BB01 - Glibenclamide - A10BB02 - Chlorpropamide - A10BB03 - Tolbutamide - A10BB09 - Gliclazide - A10BB12 - Glimepiride - A10BB31 - Acetohexamide - A10BD03 - Metformin and Rosiglitazone - A10BD04 - Glimepiride and Rosiglitazone - A10BD07 - Metformin and Sitagliptin - A10BD10 - Metformin and Saxagliptin - A10BD11 - Metformin and Linagliptin | - A10BF01 - Acarbose - A10BG02 - Rosiglitazone - A10BG03 - Pioglitazone - A10BH01 - Sitagliptin - A10BH03 - Saxagliptin - A10BH05 - Linagliptin - A10BJ01 - Exenatide - A10BJ02 - Liraglutide - A10BK01 - Dapagliflozin - A10BK02 - Canagliflozin - A10BK03 - Empagliflozin - A10BX02 - Repaglinide - A10BX03 –Nateglinide |
| --- | --- |

Source: <http://mchp-appserv.cpe.umanitoba.ca/viewConcept.php?conceptID=1085>

**Mood and Anxiety Disorder**

- one or more hospitalizations with a diagnosis of depression, episodic mood disorders (i.e., bipolar disorder, manic episode), or anxiety (i.e., anxiety disorders, phobic disorders, obsessive–compulsive disorders): ICD–9–CM codes 296.1–296.8, 300.0, 300.2–300.4, 300.7, 309, 311; ICD–10–CA codes F31, F32, F33, F34.1, F38.0, F38.1, F40, F41.0–F41.3, F41.8, F41.9, F42, F43.1, F43.2, F43.8, F45.2, F53.0, F93.0;
- one or more hospitalizations or physician visits with a diagnosis for anxiety, dissociative, and somatoform disorders: ICD-9-CM code 300; ICD-10-CA codes F41, F44, F45.0, F45.1, F48, F68.0, or F99 AND one or more prescriptions for an antidepressant or mood stabilizer, including medications with the ATC codes N06A, N05BA, N05AN01;
- one or more physician visits with a diagnosis of depression or episodic mood disorders: ICD–9–CM codes 296, 311;
- three or more physician visits with a diagnosis of anxiety, dissociative, and somatoform disorders or adjustment reaction, ICD–9–CM codes 300 and 309.

Drugs to treat mood and anxiety disorders include:

1. Antidepressants, ATC code N06A

2. Benzodiazepine Derivatives Anxiolytics, ATC code N05BA

3. Lithium, ATC code N05AN01

Source: <http://mchp-appserv.cpe.umanitoba.ca/viewConcept.php?conceptID=1391>
